# Single-Construct Polycistronic Doxycycline-Inducible Vectors Improve Direct Cardiac Reprogramming and Can Be Used to Identify the Critical Timing of Transgene Expression

**DOI:** 10.3390/ijms18081805

**Published:** 2017-08-19

**Authors:** Tomohiko C. Umei, Hiroyuki Yamakawa, Naoto Muraoka, Taketaro Sadahiro, Mari Isomi, Sho Haginiwa, Hidenori Kojima, Shota Kurotsu, Fumiya Tamura, Rina Osakabe, Hidenori Tani, Kaori Nara, Hiroyuki Miyoshi, Keiichi Fukuda, Masaki Ieda

**Affiliations:** 1 Department of Cardiology, Keio University School of Medicine, 35 Shinanomachi, Shinjuku-ku, Tokyo 160-8582, Japan; tumei@keio.jp (T.C.U.); roroyama@gmail.com (H.Y.); naotomuraoka@gmail.com (N.M.); taket0901@hotmail.co.jp (T.S.); isomi@a2.keio.jp (M.I.); s.h.lock1986@a6.keio.jp (S.H.); hideno.way@gmail.com (H.K.); krt-da-kgp@a6.keio.jp (S.K.); hyoitoanakaratokagekayo@gmail.com (F.T.); nari3969@gmail.com (R.O.); ta.hidenori@gmail.com (H.T.); knara@keio.jp (K.N.); kfukuda@a2.keio.jp (K.F.); 2 Department of Physiology, Keio University School of Medicine, 35 Shinanomachi, Shinjuku-ku, Tokyo 160-8582, Japan; hiromiyoshi@keio.jp

**Keywords:** reprogramming, cardiomyocyte, doxycycline-inducible, cell cycle, fibroblast

## Abstract

Direct reprogramming is a promising approach in regenerative medicine. Overexpression of the cardiac transcription factors Gata4, Mef2c, and Tbx5 (GMT) or GMT plus Hand2 (GHMT) directly reprogram fibroblasts into cardiomyocyte-like cells (iCMs). However, the critical timing of transgene expression and the molecular mechanisms for cardiac reprogramming remain unclear. The conventional doxycycline (Dox)-inducible temporal transgene expression systems require simultaneous transduction of two vectors (pLVX-rtTA/pLVX-cDNA) harboring the reverse tetracycline transactivator (rtTA) and the tetracycline response element (TRE)-controlled transgene, respectively, leading to inefficient cardiac reprogramming. Herein, we developed a single-construct-based polycistronic Dox-inducible vector (pDox-cDNA) expressing both the rtTA and TRE-controlled transgenes. Fluorescence activated cell sorting (FACS) analyses, quantitative RT-PCR, and immunostaining revealed that pDox-GMT increased cardiac reprogramming three-fold compared to the conventional pLVX-rtTA/pLVX-GMT. After four weeks, pDox-GMT-induced iCMs expressed multiple cardiac genes, produced sarcomeric structures, and beat spontaneously. Co-transduction of pDox-Hand2 with retroviral pMX-GMT increased cardiac reprogramming three-fold compared to pMX-GMT alone. Temporal Dox administration revealed that Hand2 transgene expression is critical during the first two weeks of cardiac reprogramming. Microarray analyses demonstrated that Hand2 represses cell cycle-promoting genes and enhances cardiac reprogramming. Thus, we have developed an efficient temporal transgene expression system, which could be invaluable in the study of cardiac reprogramming.

## 1. Introduction

Heart disease is a leading cause of death worldwide. The generation of cardiomyocytes from pluripotent stem cells (PSCs), or through a direct reprogramming approach, has great potential for the development of new treatments, including uses in disease modeling, drug discovery, and regenerative medicine [1,2,3]. We first reported that overexpression of the cardiac transcription factors Gata4, Mef2c, and Tbx5 (GMT) can directly reprogram mouse fibroblasts into functional cardiomyocyte-like cells (iCMs) without the need for a PSC-like state [4]. Since our discovery, multiple groups, including ours, have shown improved direct cardiac reprogramming through the modification of transcription factors, miRNAs, small molecules, epigenetic factors, and cell culture conditions [2,5,6,7,8,9,10,11]. Song et al. reported that the addition of Hand2, another cardiac-enriched transcription factor, to GMT (GHMT) promotes cardiac reprogramming and enhances the generation of beating iCMs in vitro [12]. Notably, the gene transfer of GMT, or GHMT, into infarct hearts reprogrammed the resident cardiac fibroblasts into iCMs in vivo and could repair infarct hearts in mice [12,13,14]. We also demonstrated that the addition of miR-133 to GMT directly inhibited Snai1, a master regulator of the epithelial-to-mesenchymal transformation, and globally suppressed fibroblast-related gene expression, allowing for an increase in cardiac reprogramming [7]. We also reported that the combination of fibroblast growth factor (FGF)-2, FGF-10, and vascular endothelial growth factor (VEGF) promoted cardiac reprogramming under serum-free cell culture conditions through the activation of Gata4, Hand2, and Nkx2.5, and through the p38 mitogen-activated protein kinase (p38 MAPK) and phosphoinositide 3-kinase/protein kinase B (PI3K/AKT) pathways [8]. However, most of these studies utilized constitutive overexpression of transgenes in fibroblasts, and thus the critical timing of transgene expression required for cardiac reprogramming, as well as the molecular mechanisms for enhancing reprogramming, remain unclear.

Dox-inducible systems have been widely used to overexpress transgenes in a temporal fashion [15]. Conventional Dox-inducible systems require the simultaneous transduction of two independent vectors—namely, reverse tetracycline transactivator (rtTA) and the tetracycline response element (TRE)-controlled transgene vectors—and subsequent Dox administration. We, and others, have previously generated two-construct-based, Dox-inducible cardiac reprogramming lentiviral vectors, consisting of pLVX-rtTA and pLVX-GMT or -GHMT [4,12,16]. However, cardiac reprogramming using these vectors was found to be inefficient, and experiments with two independent vectors are complicated and labor-intensive. 

In this study, we developed a new single-construct-based, poly-cistronic Dox-inducible lentiviral vector, expressing both the rtTA and TRE-controlled cardiac reprogramming factors. Our new polycistronic Dox-inducible vector increased the reprogramming of mouse fibroblasts into functional iCMs and promoted cardiac reprogramming to a higher degree than the conventional method. We also revealed the critical timing of exogenous transgene expression required for efficient cardiac reprogramming and identified previously unrecognized molecular events during cardiac reprogramming.

## 2. Results

### 2.1. New Polycistronic Dox-Inducible Vectors Improve Direct Cardiac Reprogramming

**Figure 1:**
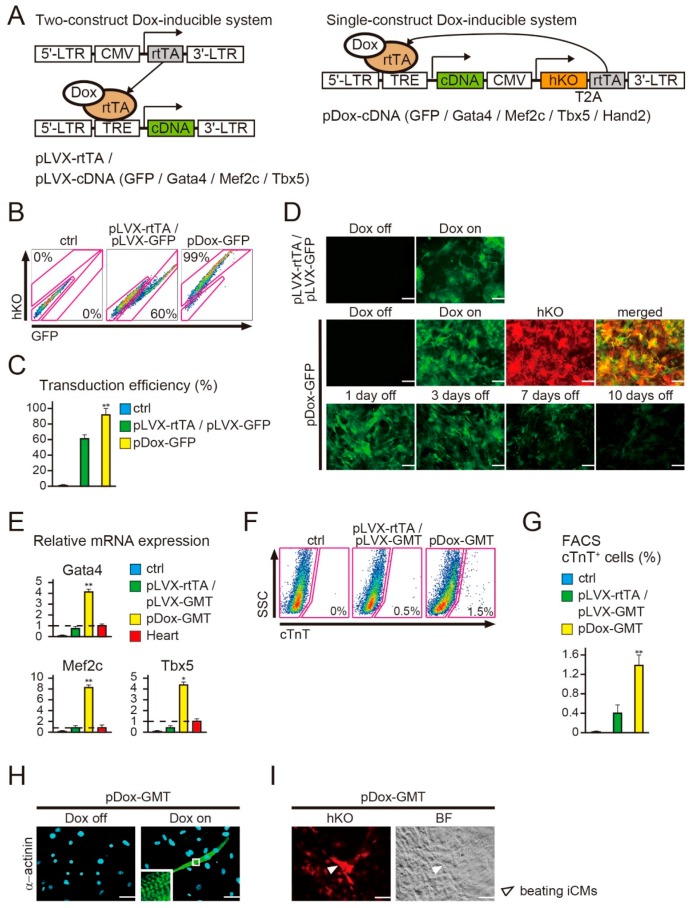
Single-construct, polycistronic doxycycline (Dox)-inducible lentiviral vectors promote cardiac reprogramming (**A**) Conventional two-construct, Dox-inducible lentiviral vectors (left, pLVX-reverse tetracycline transactivator (rtTA) and pLVX-cDNA (green fluorescent protein (GFP), Gata4, Mef2c, Tbx5)), and the new single-construct, polycistronic Dox-inducible lentiviral vectors (right, pDox-cDNA (GFP, Gata4, Mef2c, Tbx5, Hand2)). Horizontal arrows indicate gene expression and diagonal arrows indicate rtTA binding to the tetracycline response element (TRE); (**B**) Fluorescence activated cell sorting (FACS) analysis of GFP or Humanized Kusabira-Orange fluorescence (hKO) expression one week after pLVX-rtTA/pLVX-GFP or pDox-GFP transduction in mouse embryonic fibroblasts (MEFs).; (**C**) quantitative data of (**B**) are shown in (*n* = 3, independent triplicate experiments); (**D**) MEFs were infected with pLVX-rtTA/pLVX-GFP or pDox-GFP and were imaged prior to (Dox off), or one week after Dox addition (Dox on), and at the indicated time points after Dox withdrawal (1, 3, 7, and 10 days off). All images were obtained using constant exposure times and identical camera settings; (**E**) qRT-PCR for Gata4, Mef2c, Tbx5 expression in MEFs, pLVX-rtTA/pLVX-GMT-transduced MEFs, and pDox-GMT-transduced MEFs cultured for one week after Dox addition, as well as in heart tissue (*n* = 3 independent triplicate experiments). Data were normalized to the values of heart tissue (the dash lines); (**F**) FACS analyses for cardiac troponin T (cTnT) expression one week after transduction, in the presence of Dox.; (**G**) quantitative data of (**F**) (*n* = 3 independent triplicate experiments); (**H**) immunocytochemistry for α-actinin in pDox-GMT-transduced MEFs cultured without or with Dox (i.e., Dox off or Dox on) for four weeks. The high-magnification view in the inset shows the sarcomeric organization; (**I**) spontaneously beating cardiomyocyte-like cells (iCMs) generated with pDox-GMT transduction, with Dox, after four weeks (arrowheads), corresponding to Movie S1. All data are presented as mean ± SD. * *p* < 0.05, ** *p* < 0.01 vs. the relevant control. The scale bars represent 100 µm.

We previously generated Dox-inducible cardiac reprogramming lentiviral vectors, encoding pLVX-rtTA and pLVX-GMT (a mixture of pLVX-Gata4, -Mef2c, and -Tbx5) [4]. However, this system required the co-transduction of two independent vectors for temporal gene expression, leading to inefficient cardiac reprogramming. To address this issue, we developed a new single-construct-based, polycistronic Dox-inducible lentiviral vector (pDox-cDNA), expressing both the rtTA and the TRE-controlled transgenes (green fluorescent protein (GFP), Gata4, Mef2c, Tbx5, and Hand2) (Figure 1A). Humanized Kusabira-Orange fluorescence (hKO) was introduced into these constructs under the control of the human cytomegalovirus immediate early promoter (CMV) and was expressed constitutively without Dox addition to identify successfully transduced cells (Figure S1A). To determine the transduction efficiency, we first transduced a mixture of pLVX-rtTA and pLVX-GFP (pLVX-rtTA/pLVX-GFP), or pDox-GFP, into mouse embryonic fibroblasts (MEFs). As demonstrated previously, the MEFs were not contaminated with cardiomyocytes or with cardiac progenitor cells, [7,8]. FACS analyses revealed that the transduction efficiency was ~60% with pLVX-rtTA/pLVX-GFP, whereas this was improved to ~99% with pDox-GFP in the absence of Dox addition (Figure 1B,C). We next determined the expression kinetics of the pDox-GFP transgene by immunohistochemistry. The majority of fibroblasts infected with pDox-GFP expressed both hKO and GFP after Dox induction, and GFP expression diminished relatively quickly following Dox withdrawal (Figure 1D and Figure S1A). We then transduced pLVX-rtTA/pLVX-GMT (a mixture of pLVX-Gata4, -Mef2c, and -Tbx5) or pDox-GMT (a mixture of pDox-Gata4, -Mef2c, and -Tbx5) into MEFs and then treated the transduced cells with Dox. After one week, quantitative RT-PCR (qRT-PCR) analyses demonstrated that GMT expression (the sum of endogenous and exogenous GMT expression) was significantly increased in MEFs transduced with pDox-GMT compared to that in MEFs with pLVX-rtTA/pLVX-GMT (Figure 1E). Concordantly, FACS analyses revealed that cardiac troponin T (cTnT) expression, a cardiomyocyte specific marker, was increased to ~1.5% in MEFs transduced with pDox-GMT compared to ~0.5% in MEFs transduced with pLVX-rtTA/pLVX-GMT (Figure 1F,G). The pDox-GMT-transduced cells expressed α-actinin, another cardiac protein, and formed distinct sarcomeric structures (Figure 1H and Figure S1B). Moreover, a subset of the iCMs beat spontaneously in culture (Figure 1I, Movie S1). These results suggest that the new polycistronic Dox-inducible lentiviral vectors directly reprogrammed these mouse fibroblasts into functional iCMs and improved cardiac reprogramming compared to the conventional Dox-inducible lentiviral systems.

### 2.2. Diminished Cardiac Reprogramming with Lentiviral pDox-GMT (a Mixture of pDox-Gata4, -Mef2c, and -Tbx5) Compared to that Seen with Retroviral pMX-GMT (a Mixture of pMX-Gata4, -Mef2c, and -Tbx5)

**Figure 2:**
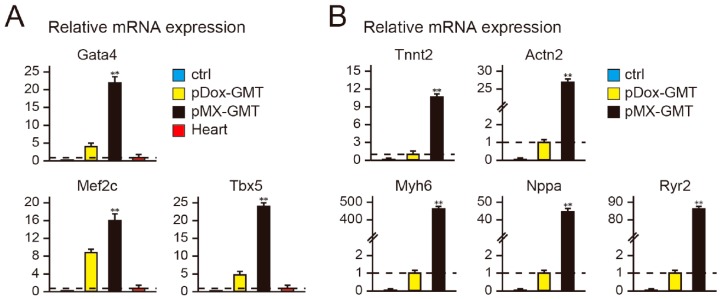

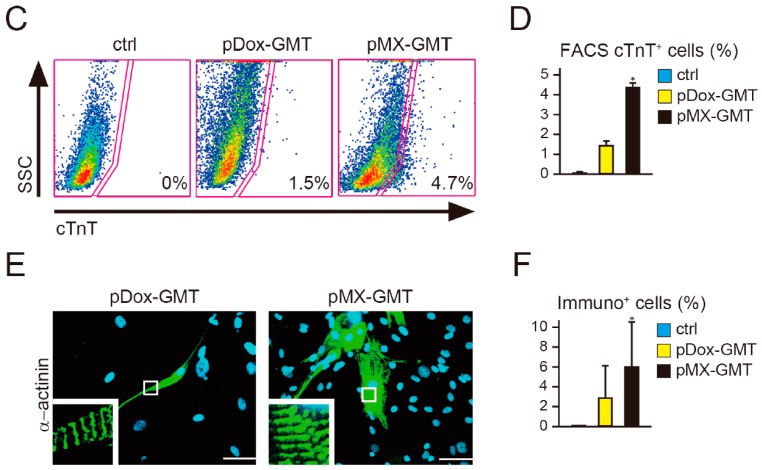
Cardiac reprogramming is more efficient using pMX-GMT (a Mixture of pMX-Gata4, -Mef2c, and -Tbx5) than that using pDox-GMT (a Mixture of pDox-Gata4, -Mef2c, and -Tbx5). (**A**) qRT-PCR for Gata4, Mef2c, and Tbx5 expression in mouse embryonic fibroblasts (MEFs), pDox-GMT-transduced MEFs, pMX-GMT-transduced MEFs, and heart tissue (*n* = 3, independent triplicate experiments). Data were normalized to the values of heart tissue (the dash lines); (**B**) qRT-PCR for TnnT2, Myh6, Nppa, and Ryr2 mRNA expression in mouse embryonic fibroblasts (MEFs), pDox-GMT-transduced MEFs and pMX-GMT-transduced MEFs after one week (*n* = 3 independent triplicate experiments). Data were normalized to the values of pDox-GMT-transduced MEFs (the dash lines); (**C**) FACS analyses of cardiac troponin T (cTnT) expression induced by pDox-GMT in the presence of Dox or pMX-GMT after one week; (**D**) quantitative data of (**C**) (*n* = 3 independent triplicate experiments); (**E**) immunocytochemistry for α-actinin in pDox-GMT-transduced MEFs in the presence of Dox, and pMX-GMT-transduced MEFs, after four weeks. The high-magnification views in the insets show the sarcomeric organization; (**F**) quantitation of α-actinin-positive cells (*n* = 3 independent triplicate experiments). All data are presented as mean ± SD. * *p* < 0.05, ** *p* < 0.01 vs. the relevant control. The scale bars represent 100 µm.

In previous cardiac reprogramming studies, we and others have principally used the retroviral vector pMX-GMT (a mixture of pMX-Gata4, -Mef2c, and -Tbx5), which robustly, and constitutively, overexpresses GMT [4,7,8,9,10,11,12,17]. It was previously unclear whether the Dox-inducible lentiviral system was as efficient as the retroviral pMX-GMT for cardiac induction [18,19]. To test this, we analyzed the relative mRNA expression of GMT (i.e., the sum of endogenous and exogenous GMT) and cardiac gene induction in MEFs transduced with pDox-GMT or pMX-GMT by qRT-PCR. pMX-GMT-transduced MEFs expressed higher levels of GMT than MEFs transduced with pDox–GMT with Dox addition (Figure 2A). Concordantly, the expression of a panel of cardiac genes related to different cardiac cell functions, including sarcomeric genes (Tnnt2, Actn2, Myh6), a cardiac hormone (Nppa), and a calcium flux-associated gene (Ryr2), were greatly induced with pMX-GMT compared to that with pDox-GMT (Figure 2B). FACS analyses also revealed that the efficiency of cTnT^+^ cell generation was 4.7% with pMX-GMT transduction, whereas it was 1.5% with pDox-GMT (Figure 2C,D). In addition, immunocytochemistry demonstrated that, after four weeks, the use of pMX-GMT generated two-fold more α-actinin^+^ cells than pDox-GMT (Figure 2E,F). Thus, although pDox-GMT was useful for studying cardiac reprogramming as it relates to temporal transgene expression, retroviral pMX-GMT was more efficient. 

### 2.3. Induction of Hand2 Transgene Expression One Day after Transduction Promotes Cardiac Reprogramming

**Figure 3:**
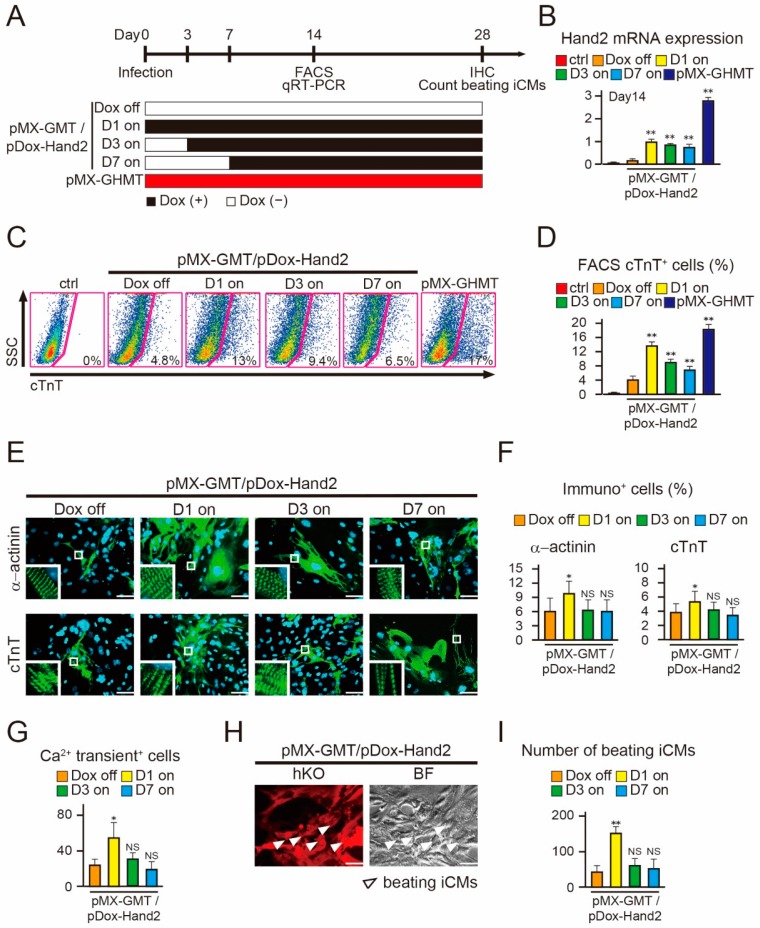
Initiating expression of Hand2 transgene one day after transduction promotes cardiac reprogramming. (**A**) Schematic representation of Hand2 transgene expression with temporal Dox addition. Mouse embryonic fibroblasts (MEFs) were infected with a mixture of pDox-Hand2 and pMX-GMT, and then Dox was added at day 1 (D1 on), day 3 (D3 on), and day 7 (D7 on) following transduction. Dox off indicates without Dox and pMX-GHMT (GMT plus Hand2) is the positive control; (**B**) qRT-PCR for Hand2 expression in the indicated samples after two weeks (*n* = 3 independent triplicate experiments); (**C**) FACS analyses of cardiac troponin T (cTnT) expression in the indicated samples after two weeks; (**D**) quantitative data of (**C**) (*n* = 3 independent triplicate experiments); (**E**) immunocytochemistry for α-actinin and cTnT after four weeks. The high-magnification views in the insets show the sarcomeric organization in cardiomyocyte-like cells (iCMs); (**F**) quantitation of the immunopositive (α-actinin^+^ and cTnT^+^) cells (*n* = 3 independent triplicate experiments); (**G**) total number of Ca^2+^ oscillation^+^ cells in ten randomly selected fields per well after four weeks (*n* = 3 independent triplicate experiments). Movie S2 shows Ca^2+^ imaging; (**H**) spontaneously beating iCMs in the pDox-Hand2/pMX-GMT-transduced MEFs after four weeks (D1 on, arrowheads), corresponding to Movie S3; (**I**) total number of beating cells in ten randomly selected fields per well (*n* = 3 independent triplicate experiments). All data are presented as mean ± SD. * *p* < 0.05, ** *p* < 0.01 vs. the relevant control; NS = not significant. The scale bars represent 100 µm.

It has been reported that the addition of pMX-Hand2 to pMX-GMT (pMX-GHMT) promotes cardiac reprogramming compared to that seen with pMX-GMT alone [8,12]. However, the optimum timing of Hand2 expression required for cardiac reprogramming was previously unclear. To address this, we next transduced a mixture of pDox-Hand2 and pMX-GMT into MEFs and administered Dox in a temporal manner. First, to determine whether Hand2 expression was necessary at the beginning of cardiac reprogramming, we induced Hand2 transgene expression at three time points: Dox addition from day 1 (D1 on), day 3 (D3 on), and day 7 (D7 on) after viral transduction (Figure 3A). Dox treatment was continued for up to a total of four weeks and pMX-GHMT was used as the positive control. Hand2 mRNA expression was significantly higher following Dox addition, and was comparable among the D1, D3, and D7 groups after fourteen days of transduction (Figure 3B). FACS analyses demonstrated that “D1 on” induced cTnT^+^ cells had an efficiency of 13%, which was three-fold higher than that without Dox (Dox off) (Figure 3C,D). Intriguingly, cTnT-expressing cells gradually decreased to 9% and 6% in the “D3 on” and “D7 on” groups, respectively. In addition, immunohistochemistry revealed that the numbers of α-actinin^+^ and cTnT^+^ cells were increased only in the “D1 on” group, but not in the “D3 on” or “D7 on” group, as compared to those in the “Dox off” group after four weeks (Figure 3E,F). Moreover, only the “D1 on” group generated a higher number of spontaneous Ca^2+^ oscillation^+^ cells and beating iCMs, compared to the “Dox off” group after four weeks (Figure 3G–I and Movie S2,S3). These results suggest that Hand2 transgene expression must be initiated one day after transduction to promote optimal cardiac reprogramming. 

### 2.4. Hand2 Transgene Expression for Two Weeks is Sufficient to Promote Cardiac Reprogramming

**Figure 4:**
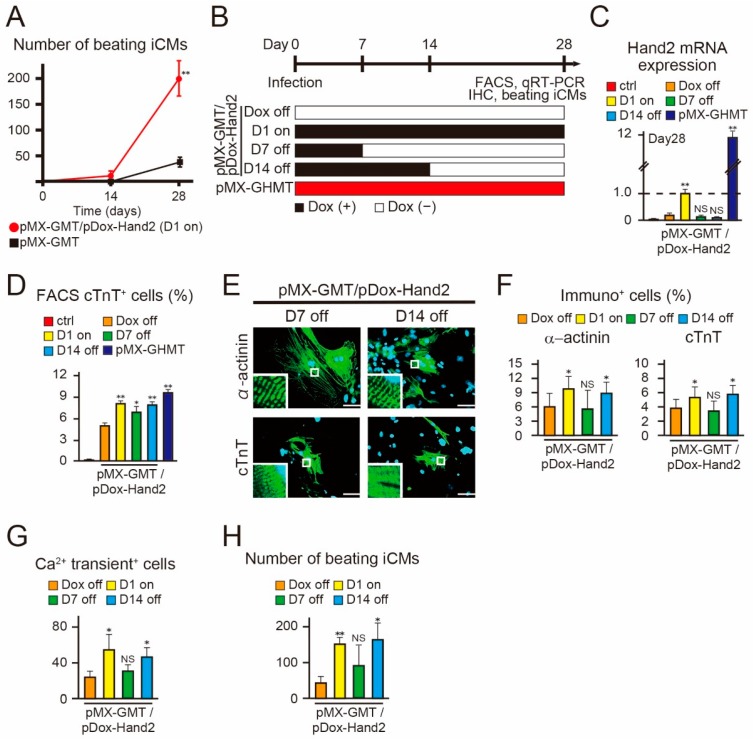
*Hand2* transgene expression for the first two weeks is sufficient to promote cardiac reprogramming. (**A**) Time course of the total number of beating cardiomyocyte-like cells (iCMs) in mouse embryonic fibroblasts (MEFs) induced with pDox-Hand2/pMX-GMT or pMX-GMT after two and four weeks (*n* = 3 independent triplicate experiments); (**B**) schematic representation of the strategy to determine the optimal timing of Hand2 expression for cardiac reprogramming. Doxycycline (Dox) was administered for different intervals, four weeks (D1 on), seven days (D7 off), and fourteen days (D14 off), after transduction of cells with pDox-Hand2/pMX-GMT; (**C**) qRT-PCR for Hand2 expression in the indicated samples after four weeks (*n* = 3 independent triplicate experiments); (**D**) FACS analyses for cardiac troponin T (cTnT) expression in the indicated samples after four weeks. Quantitative data are shown (*n* = 3 independent triplicate experiments); (**E**) immunocytochemistry for α-actinin and cTnT after four weeks. The high-magnification views in the insets show the sarcomeric organization; (**F**) quantitation of immunopositive (α-actinin^+^ and cTnT^+^) cells (*n* = 3 independent triplicate experiments); (**G**) total number of Ca^2+^ oscillation^+^ cells in ten randomly selected fields per well after four weeks (*n* = 3 independent triplicate experiments); (**H**) total number of spontaneously beating cells ten randomly selected fields per well after four weeks (*n* = 3 independent triplicate experiments). All data are presented as mean ± SD. * *p* < 0.05, ** *p* < 0.01 vs. the relevant control; NS = not significant. The scale bars represent 100 µm.

We found that, in the presence of Hand2 transgene expression, the number of beating iCMs greatly increased during the last two weeks of cardiac reprogramming (Figure 4A). We next investigated the duration of Hand2 transgene expression sufficient to promote cardiac reprogramming after four weeks. We transduced pMX–GMT/pDox–Hand2 into MEFs, administered Dox for different intervals, specifically for four weeks (D1 on), for seven days (D7 off), and for fourteen days (D14 off) after transduction, and determined the cardiac reprogramming efficiency (Figure 4B). qRT–PCR demonstrated that after four weeks, Hand2 mRNA expression was decreased in the “D7 off” and “D14 off” groups to levels similar to those observed in the “Dox off” group, whereas expression was maintained in the “D1 on” and pMX–GHMT groups (Figure 4C). Intriguingly, FACS analyses demonstrated that the induction of cTnT^+^ cells was elevated and comparable between the “D14 off” and “D1 on” cells after four weeks (Figure 4D). Immunohistochemistry for α-actinin and cTnT expression revealed that the “D14 off” condition increased iCM generation to a similar as level as that seen with the “D1 on” condition (Figure 4E,F). Moreover, spontaneous Ca^2+^ oscillation^+^ cells and beating iCMs were increased in the “D14 off” and “D1 on” groups, but not in the “D7 off” group (Figure 4G,H). These results suggest that exogenous Hand2 expression for the first two weeks is sufficient to promote cardiac reprogramming and generate beating iCMs after four weeks.

### 2.5. Hand2 Activates a Cardiac Program and Represses Cell Cycle-Promoting Genes During Cardiac Reprogramming 

**Figure 5:**
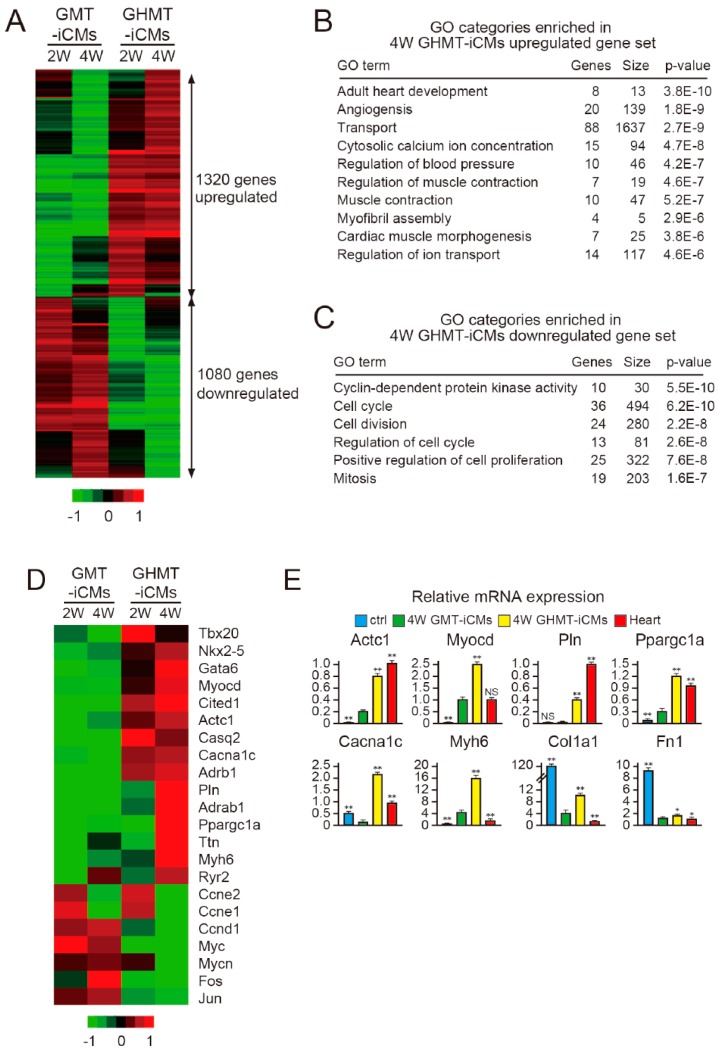
Hand2 represses cell cycle-promoting genes during cardiac reprogramming. (**A**) Microarray analyses of FACS-sorted α myosin heavy chain (αMHC)-GFP^+^ cells induced with pMX-GMT or pMX-GHMT after two (2W) and four weeks (4W). Red indicates increased gene expression, whereas green indicates decreased gene expression; (**B**) gene ontology (GO) term analyses for the upregulated genes in GHMT–iCMs (cardiomyocyte-like cells) compared to expression in GMT-iCMs after four weeks. Cardiac-related GO categories are shown; (**C**) GO term analyses for the downregulated genes in GHMT-iCMs compared to expression in GMT-iCMs after four weeks. Cell cycle-related GO categories are shown; (**D**) heatmap images of the gene expression patterns of cardiac- and cell cycle-related genes; (**E**) the relative mRNA expression of the indicated genes was determined by qRT-PCR (*n* = 3 independent triplicate experiments). All data are presented as mean ± SD. * *p* < 0.05, ** *p* < 0.01 vs. the relevant control. The scales are −1 to 1 in log2.

Hand2 promotes direct reprogramming of highly proliferative fibroblasts into post-mitotic iCMs; however, the molecular mechanisms associated with this were previously unclear. To investigate the effects of Hand2 on cardiac reprogramming, we performed microarray analyses to determine the transcriptional profiles of iCMs induced with pMX-GMT and pMX-GHMT. We used α myosin heavy chain (MHC)-GFP mice, in which only the cardiomyocytes express GFP, transduced the MEFs with pMX-GMT or pMX-GHMT, and isolated αMHC-GFP^+^ iCMs by FACS after two and four weeks [4]. Microarray analyses revealed that the addition of Hand2 to GMT resulted in the upregulation of 1320 genes and the downregulation of 1080 genes after two and four weeks, suggesting that both positive and negative gene regulation might play roles in Hand2-mediated cardiac reprogramming (Figure 5A). Gene ontology (GO) term analyses revealed that genes related to the cardiac program, such as heart development, muscle contraction, and regulation of ion transport, were highly enriched in the upregulated gene set, whereas genes related to the cell cycle including cell division, cell proliferation, and mitosis were highly enriched in the downregulated gene set (Figure 5B,C). Heat-map analyses and qRT-PCR confirmed that the addition of Hand2 upregulates a panel of cardiac genes including cardiac transcription factors (*Tbx20*, *Nkx2.5*, *Myocd*, *Gata6*), ion channel-related genes (*Casq2*, *Cacna1c*, *Pln*, *Ryr2*), one mitochondrial gene (*Ppargc1a*), and several sarcomeric genes (*Actc1*, *Myh6*, *Ttn*). Intriguingly, Hand2 expression suppressed multiple cell cycle-promoting genes such as *Ccne1*/*2*, *Ccnd1*, *Myc*, *Mycn*, *Fos*, and *Jun*, but not fibroblast-related genes including *Col1a1* and *Fn1* (Figure 5D,E). These results suggest that Hand2 addition activates a cardiac program and concomitantly represses cell cycle-promoting genes to reprogram fibroblasts into iCMs.

## 3. Discussion

In this study, we developed a new single-construct-based, polycistronic Dox-inducible cardiac reprogramming vector that expressed both the rtTA and TRE-controlled transgenes. The new Dox-inducible vectors reprogrammed mouse fibroblasts into functional iCMs and improved cardiac reprogramming compared to the conventional two-construct-based, Dox-inducible lentiviral systems. We also identified the critical timing of Hand2 transgene expression required for cardiac reprogramming, and demonstrated that Hand2 suppresses cell-cycle-promoting genes during this process.

We, and others, have previously used two-vector-based, Dox-inducible lentiviral systems to express cardiac reprogramming factors [4,12,16]. Although we were successful in generating iCMs that expressed cardiac genes and produced sarcomeric structures by co-transducing pLVX-rtTA and pLVX-GMT vectors, the generation of beating iCMs was inefficient [4,18,19]. To improve the reprogramming efficiency and mitigate the laborious procedure of generating multiple vectors, we developed a single-vector-based, polycistronic Dox-inducible lentiviral system. pDox-GMT resulted in the formation of spontaneously beating iCMs from MEFs and more efficiently reprogrammed fibroblasts into iCMs, compared to the conventional Dox-inducible vectors. Although we generated beating iCMs using pDox-GMT in this study, we did not directly demonstrate the electrophysiological properties of these iCMs. It is therefore important, in the future, to determine whether the constant forced expression of the GMT factors leads to any electrophysiological abnormalities in these iCMs. We also found that retroviral pMX-GMT was more efficient for cardiac reprogramming than lentiviral pDox–GMT. Differences in the viral particles, the Dox-inducible gene expression system, and the stoichiometry of reprogramming factors might all contribute to the lower cardiac reprogramming efficiency seen with pDox-GMT [17,18,20]. However, it should be noted that the Dox-inducible fibroblast cell line might improve cardiac reprogramming [21].

We also demonstrated that constitutive expression of Hand2 with GMT for four weeks improved GMT-mediated cardiac reprogramming; however, it was important to understand at what time is Hand2 expression required to improve reprogramming. Using pDox-Hand2, in the presence of constitutive GMT expression, we demonstrated for the first time that Hand2 must be expressed from day one of cardiac reprogramming in order for it to improve cardiac reprogramming efficiency. In the developing heart, Hand2 and GMT are expressed at the same developmental stage [22], and Hand2 physically interacts with GMT in order to activate cardiac gene expression [23,24,25]. Thus, it is conceivable that GHMT might form a transcription factor complex to promote cardiac reprogramming. We also found that two weeks of Hand2 transgene expression is sufficient to generate beating iCMs and promote cardiac reprogramming after four weeks. These results are consistent with previous reports demonstrating that two weeks of GMT or GHMT expression can induce cardiac gene expression and Ca^2+^ transient^+^ cells in fibroblasts [4,12,16]. However, previous studies did not examine the optimal time for initiation of transgene expression, nor the generation of beating iCMs, a more important characteristic of differentiated cardiomyocytes. Thus, our findings advance on the previous results and demonstrate functional cardiac reprogramming stability, which requires two weeks of transgene expression.

Importantly, we also identified unprecedented transcriptional changes induced by Hand2 during cardiac reprogramming. Hand2 suppressed multiple cell cycle-promoting genes and enhanced cardiac reprogramming. In contrast, the addition of cell cycle-promoting genes such as *Myc* have been shown to improve the generation of induced PSCs (iPSCs), suggesting that the reprogramming mechanisms might be distinct between iCM and iPSC generation [26,27]. Fibroblasts and iPSCs are proliferative cell types, whereas cardiomyocytes are terminally differentiated post-mitotic cells. Thus, it might be expected that iCM reprogramming would be negatively correlated with cell proliferation and the expression of cell cycle genes.

In summary, we have developed a new single-construct-based, polycistronic Dox-inducible lentiviral vector that improves cardiac reprogramming. This new system should be a valuable platform for understanding the mechanisms of cardiac reprogramming in basic and pre-translational research, and also might be applicable to other fields that utilize direct reprogramming. 

## 4. Materials and Methods

### 4.1. Plasmid Construction

We generated the Dox-inducible lentiviral vectors, pDox–GFP, pDox–GMT, and pDox–Hand2, by subcloning cDNAs into the CSIV-TRE-RfA-CMV-KT (RIKEN BSI) using the Gateway system (Thermo Fisher Scientific, Waltham, MA, USA). Gata4, Hand2, Mef2c, Tbx5, and GFP cDNAs were subcloned from the pMX vectors as described previously [4].

### 4.2. Generation of α Myosin Heavy Chain (αMHC)-Green Fluorescent Protein (GFP) Mice

Transgenic mice overexpressing GFP under the αMHC promoter were generated as described previously [4]. The Keio University Ethics Committee for Animal Experiments approved all experiments in this study.

### 4.3. Mouse Embryonic Fibroblast (MEF) Isolation

For MEF isolation, embryos were harvested from 12.5-day pregnant mice, washed with PBS, and following decapitation, the visceral tissues, including the heart were removed [7]. The remaining parts of the embryos were minced, then transferred to a 0.125% trypsin/Ethylenediaminetetraacetic acid (EDTA) solution (Thermo Fisher Scientific, 25200-072, 3 mL per embryo), and incubated at 37 °C for 20 min. An additional 3 mL of trypsin/EDTA solution was then added, and the mixture was further incubated at 37 °C for 20 min. After trypsinization, 6 mL of Dulbecco’s Modified Eagle Medium (DMEM) containing 10% Fetal Bovine Serum (FBS) was added and pipetted several times to allow tissue dissociation. After incubation of the tissue/medium mixture for 5 min at room temperature, the supernatant was transferred to a fresh tube, and cells were collected by centrifugation and resuspended in DMEM/10% FBS (Thermo Fisher Scientific, SV30014.03) for culturing at 37 °C in 5% CO_2_. The explants were plated on 100 mm gelatin-coated dishes at a ratio of one embryo per 2–3 dishes, then cultured in explant medium (Iscove’s Modified Dulbecco’s Medium (IMDM) with l-glutamate and 25 mM HEPES (Thermo Fisher Scientific, 12440-053)/20% FBS), and passaged 1:3 for further expansion (passage 1). For all experiments, fibroblasts were used at passages 1–3. Fibroblasts that migrated were harvested and filtered with 40 µm cell strainers (BD Biosciences, San Jose, CA, USA) to avoid contamination with tissue fragments. The cells were plated at a density of 1 × 10^4^ cells/cm^2^ for retrovirus and lentivirus transduction.

### 4.4. Lentivirus Production

293T cells were plated at a density of 5 × 10^6^ cells per 100 mm plate in 10 mL of medium containing Dulbecco’s modified Eagle’s medium (Wako, Tokyo, Japan, 044-29765), and incubated for 24 h at 37 °C in 5% CO_2_. On the following day, we prepared a total volume of 450 µL of plasmid DNA solution containing 17 µg of the pDox-cDNA, 10 µg of the packaging plasmid (pMDLg/pRRE), and 10 µg of the vesicular stomatitis virus glycoprotein (VSV-G) and Rev-expressing plasmid (pCMV-VSV-G-RSV-Rev) in a 5 mL polystyrene tube, after which 50 µL of 2.5 M CaCl_2_, and 500 µL of 2× BBS (50 mM BES, NaCl 280 mM, Na_2_HPO_4_ 1.5 mM) were added. After incubation for 10–20 min at room temperature, the mixture was transferred to a 100 mm plate of 293T cells (75% confluency), followed by incubation for 24 h at 37 °C in 5% CO_2_. The medium was replaced with 7.5 mL of DMEM containing 10 µM Forskolin (Sigma Aldrich, St. Louis, MO, USA , F6886), and then incubated for 24 h at 37 °C in 5% CO_2_. 

### 4.5. Retroviral and Lentiviral Infection 

The pMX retroviral vectors were generated as described previously [4]. Fibroblasts were transduced with retrovirus and lentivirus vectors with 4 µg/mL polybrene (Millipore, Billerica, MA, USA , TR-1003-G). The medium was replaced with FGF-2, FGF-10, and VEGF (FFV) medium after 24 h of infection as described previously [8]. FFV medium contained StemPro-34 SF medium (Thermo Fisher Scientific, 10639-011)/GlutaMAX (10 µL/mL, Thermo Fisher Scientific, 35050-061)/ascorbic acid (50 µg/mL, Sigma Aldrich, A-4544)/recombinant human VEGF165 (5 ng/mL, R&D Systems, Minneapolis, MN, USA, 293-VE-050)/recombinant human basic FGF (146 aa) (10 ng/mL, R&D Systems, 233-FB-025)/recombinant human FGF-10 (50 ng/mL, R&D Systems, 345-FG-025). The medium was changed every 2–3 days. Dox was administered as indicated at 1 µg/mL for gene induction. 

### 4.6. Fluorescence Activated Cell Sorting (FACS) Analyses and Sorting 

For cTnT expression, cells were fixed with 4% paraformaldehyde (PFA; Muto Pure Chemicals, Tokyo, Japan, 3311-1) for 15 min, permeabilized with saponin (Sigma Aldrich, 47036-250G-F), stained with an anti-cTnT antibody (Thermo Scientific, MS-295-P1), followed by a secondary antibody conjugated with Alexa Fluor 488 or 647 (Thermo Fisher Scientific). Cells were then analyzed using a FACS Calibur instrument (BD Biosciences) with the FlowJo software (Tomy Digital Biology, Tokyo, Japan). For iCM sorting, cells were sorted as αMHC-GFP^+^ cells [4]. The negative controls used for FACS gating were based on the cells stained with isotype control antibodies. 

### 4.7. Quantitative RT-PCR 

Total RNA was isolated from the cells using the RNA Cell Miniprep System (Promega, Madison, WI, USA, Z6012), and qRT–PCR was performed using the StepOnePlus Real-Time PCR system with the following TaqMan probes (Thermo Fisher Scientific): *Gata4* (Mm00484689_m1), *Mef2c* (Mm01340842_m1), *Tbx5* (Mm00803518_m1), *Tnnt2* (Mm00441922_m1), *Actn2* (Mm00473657_m1), *Myh6* (Mm00440354_m1), *Nppa* (Mm01255747_g1), *Ryr2* (Mm00465877_m1), *Hand2* (Mm00439247_m1), *Actc1* (Mm01333821_m1), *Myocd* (Mm00455051_m1), *Pln* (Mm00452263_m1), *Ppargc1* (Mm01208835_m1), *Cacna1c* (Mm00437917_m1), *Col1a1* (Mm00801666_g1), and *Fn1* (Mm01256744_m1). mRNA levels were normalized to those of *Gapdh* (Mm99999915_g1). 

### 4.8. Immunocytochemistry 

Immunostaining was performed as previously described [8]. Briefly, cells were fixed in 4% PFA for 15 min at room temperature, blocked with 5% Normal Goat Serum Blocking Solution (Vector Laboratories, Burlingame, CA, USA, S-1000). The following primary antibodies were used: mouse anti-cTnT (Thermo Fisher Scientific, MS-295-P1); mouse anti-sarcomeric α-actinin (Sigma Aldrich, 111M4845). Cells were then incubated with secondary antibodies conjugated with Alexa Fluor 488, followed by DAPI (Thermo Fisher Scientific, D1306) counterstaining. The numbers of cells immunopositive for cTnT and α-actinin were counted in all fields per well from at least three independent experiments. The measurements and calculations were conducted in a blinded manner.

### 4.9. Ca^2+^ Imaging and Counting Beating Cells 

Ca^2+^ imaging was performed following standard protocols [8]. Briefly, cells were labeled with Rhod-3 from a Calcium Imaging Kit (Thermo Fisher Scientific, R10145) for 1 h at room temperature, washed, and incubated for an additional hour, to allow de-esterification of the dye. Rhod-3-labeled cells were analyzed at 37 °C using an LSM 510 META confocal microscope (Carl Zeiss, Oberkochen, Germany). Ca^2+^ oscillation^+^ cells were counted in ten randomly selected fields per well in at least three independent experiments. To count the number of beating cells, we seeded 50,000 fibroblasts per well on 12-well plates, performed cell transductions, cultured the cells with the indicated media, and then monitored cell contraction. For accurate analyses of the cell count, we used an all-in-one fluorescence microscope as described previously (BZ-9000; Keyence, Tokyo, Japan) [8]. We first acquired images of the cells in all the areas in a well with a 20× phase contrast lens by moving the motorized stage sequentially. We next moved the field to cover all the areas in a well, and counted the number of spontaneously contracting cells in each field with the 20× phase contrast lens in at least three independent experiments. The measurements and calculations were conducted in a blinded manner.

### 4.10. Microarray Analyses 

Genome-wide gene expression analyses were performed using the 3D-Gene Mouse Oligo Chip 25k (Toray Industries Inc., Tokyo, Japan) as described previously [7]. RNA was extracted from pMX-GMT or pMX-GHMT-induced αMHC-GFP^+^ cells, sorted using a FACS Aria III instrument, using the ReliaPrep RNA Cell Miniprep System (Promega, Z6012) at Day 14 and Day 28. The raw data for each spot were normalized by substitution with the mean intensity of the background signal determined by the combined signal intensities of all blank spots at 95% confidence intervals. Raw data intensities greater than 2 SDs of the background signal intensity were considered valid. Signals detected for each gene were normalized by the global normalization method. Heat-map images for differentially expressed genes (more than 2-fold) were processed using Cluster (v.2.0) software (http://bonsai.hgc.jp/~mdehoon/software/cluster/software.htm). Scatter plot analyses and GO analyses were performed as described previously [7]. The accession number for the microarray data reported in this paper is NCBI GEO:GSE101578.

### 4.11. Statistical Analyses 

Differences between groups were examined for statistical significance by Student’s *t*-tests or analysis of variance (ANOVA). Differences with *p* values of less than 0.05 were regarded as significant. * *p* < 0.05; ** *p* < 0.01.

## Supplementary Materials

Supplementary materials can be found at www.mdpi.com/1422-0067/18/8/1805/s1.
