# Supplementary material for: Single-Construct Polycistronic Doxycycline-Inducible Vectors Improve Direct Cardiac Reprogramming and Can Be Used to Identify the Critical Timing of Transgene Expression
Source: Int J Mol Sci. 2017 Aug 19;18(8):1805. doi: 10.3390/ijms18081805 (PMC5578192; doi:10.3390/ijms18081805)
Supplement: Supplementary file 1 [file ijms-18-01805-s001.zip › ijms-217415 supplementary/Umei_supple_legends.docx]

**Supplementary**


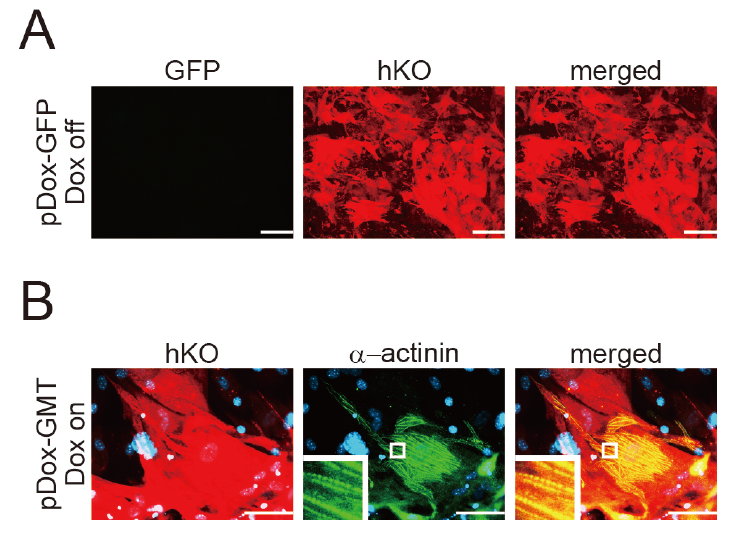


**Figure S1.** Related to Figure 1. Single-construct, polycistronic doxycycline (Dox)-inducible lentiviral vectors promote cardiac reprogramming. (A) MEFs were transduced with pDox-GFP and imaged for GFP and hKO expression without Dox addition; (B) Immunocytochemistry for α-actinin and hKO in pDox-GMT-transduced MEFs cultured with Dox for four weeks. The high-magnification view in the inset shows organization of the sarcomere. The scale bars represent 100 µm.

**Supplementary Movie Legends**

**Movie S1.** Related to Figure 1I. Spontaneously beating pDox-GMT-induced iCMs cultured for four 4 weeks in the presence of Dox.

**Movie S2.** Related to Figure 3G. Spontaneous Ca^2+^ oscillations in pDox-Hand2/pMX-GMT-induced iCMs cultured for four weeks in the presence of Dox.

**Movie S3.** Related to Figure 3H. Spontaneously beating pDox-Hand2/pMX-GMT-induced iCMs cultured for four weeks in the presence of Dox.
